# Wave exposure reduces herbivory in post-disturbed reefs by filtering species composition, abundance and behaviour of key fish herbivores

**DOI:** 10.1038/s41598-020-66475-y

**Published:** 2020-06-19

**Authors:** Rucha Karkarey, Pooja Rathod, Rohan Arthur, Shreya Yadav, Anne Theo, Teresa Alcoverro

**Affiliations:** 1 Nature Conservation Foundation, 3076/5, IV Cross, Gokulam Park, Mysore 570002 India; 2 Centre d’Estudis Avançats de Blanes (CEAB, CSIC). Dept. of Marine Ecology. Accés a la Cala S. Francesc 14.17300 Blanes, Girona, Spain; 3 National Centre for Biological Sciences (NCBS), Tata Institute of Fundamental Research (TIFR), Bangalore, 560 065 India; 4 University of Hawaii, Manoa, USA; 5 Centre for Ecological Sciences (CES), Indian Institute of Science (IISc), Bangalore, 560012 India

**Keywords:** Ecology, Ecology, Ocean sciences

## Abstract

Harsh environmental conditions limit how species use the landscape, strongly influencing the way assemblages are distributed. In the wake of repeated coral bleaching mortalities in Lakshadweep, we examined how wave exposure influences herbivory  in exposed and sheltered reefs. We used a combination of i. field observations of fish herbivore composition, abundance and activity across 6 exposed and 6 sheltered reefs; ii. experimental manipulations in a subset of these reefs (herbivore exclosures); and iii. opportunistic observations of fish recruitment, to determine how exposure influences herbivore biomass and herbivory. Species richness, biomass, abundance, total bite rates and species-specific per capita bite rates were lower in exposed compared to sheltered reefs, linked to strong environmental filtering of species composition, abundance and behaviour. For some critical species, this environmental filtering begins with differential recruitment and post-recruitment processes between exposures. Bite rates at sheltered sites were dominated by just a few species, most being laterally compressed surgeonfish that may find it difficult accessing or surviving in wave-battered shallow reefs. Exclosure experiments confirmed  that exposed reefs  were less controlled by herbivores than sheltered reefs. In post-disturbed reefs like Lakshadweep, environmental gradients appear to be key mediators of critical functions like herbivory by determining species composition, abundance and behaviour.

## Introduction

At harsh environmental extremes, most biotic interactions are likely overwhelmed by species’ abilities to cope with challenging conditions. Since not all species can survive extreme conditions, communities may be more abiotically assembled as less tolerant species drop out. Filtering by the environment appears to be widespread, shaping assemblages as widely different as bacteria, fungi, plants, birds and bees, among others^1–6^. In addition, for the species that do persist, several modifications in behaviour and physiology may occur, often with longer-term life-history and demographic consequences. Sessile organisms may respond with morphological changes (e.g. increased anchorage systems, modified structural forms, change in leaf morphology) that allow them to withstand physical forces like wind, desiccation, wave exposure, etc.^7–9^. Mobile species may change how they use the landscape, modifying their morphology, movement or foraging behaviour to persist in high wind or wave swept locations^10,11^.

How species navigate environmental conditions can have major consequences for the way functions (such as herbivory and predation) are distributed across an ecosystem. A selective reduction in species richness and population abundance, together with reduced foraging and movement across the landscape, can result in a potential weakening of interaction strengths, including critical trophic functions that prevail in less constrained conditions^12,13^. By limiting critical ecosystem functions, abiotic forces may place natural limits on ecosystem resilience in harsh environmental regimes.

Where herbivory is a primary driver of ecosystem regulation, understanding how abiotic forcing can influence its impact may be essential to determining ecosystem resilience. In coral reefs, herbivory by fish and sea urchins is a central agent of system health, mediating competitive interactions between coral and algae^14,15^. The strength of herbivory is critical in the wake of major coral mortalities, when benthic recovery is heavily predicated on maintaining reefs algal free^16–18^. Understanding how the capacity of herbivores to control algal production varies with different environmental regimes can help qualify how likely different reefscapes are to recover from catastrophic coral mortalities.

By influencing the underlying reef habitat, exposure can mediate the assemblage of species that are able to recruit to^19^, survive in^20^, and effectively use these environments. After a disturbance, hydrodynamic forces affect the rate at which new coral habitat is formed and old coral habitat degrades^21,22^. Habitat structure-dependent fish species are most likely to be affected by these changes^23^, and many long-lived benthic species will remain restricted to only the least dynamic, most stable sites^24^. Physical exposure gradients also filter fish species based on their form and swimming traits^25^; turbulent environments are known to limit the feeding function of laterally compressed fish body forms and favour rounder or fusiform body shapes^26^. Algal growth can also be strongly influenced by physical exposure gradients: productivity may increase up to a point, before declining again as drag and dislodgement forces overwhelm growth^27^. Whether herbivores can compensate for differences in algal growth along this gradient is far from certain. The capacity of the assemblage to control algal production will be highly contingent on how strongly physical exposure filters herbivore assemblages, abundance and feeding activity on the one hand, and how it filters algal assemblages and growth on the other.

The coral reefs of Lakshadweep Archipelago are an ideal natural laboratory to examine how physical exposure influences fish herbivory in reefs under recovery conditions. The atolls have strong exposure contrasts in relation to the prevailing southwest monsoon. The archipelago has been subject to repeated coral mass mortalities, recording losses of 87% and 44% of live coral cover in the wake of the 1998 and 2010 bleaching events^28^. The high density of fish herbivores, together with coral recruitment ensured a remarkable recovery after the 1998 bleaching event^29^, although recovery after 2010 was more protracted because of limited recruitment and survival of fast-growing coral^28^. Importantly, until very recently, these reefs remain lightly fished, with the commercial fishery targeting pelagic stocks^24^. This allowed us to examine how wave exposure influences the ability of herbivore assemblages to control algal growth at three atolls in the Lakshadweep without having to account for fishing. We used a combination of comparative field studies - between sheltered and exposed reefs - of herbivore fish composition, abundance and activity (underwater filming to measure herbivory), experimental manipulations (herbivore exclosures), and opportunistic observations of fish recruitment to determine how wave exposure influences the effectiveness of herbivory in controlling algal production.

## Materials and methods

### Study area and design

**Figure 1:**
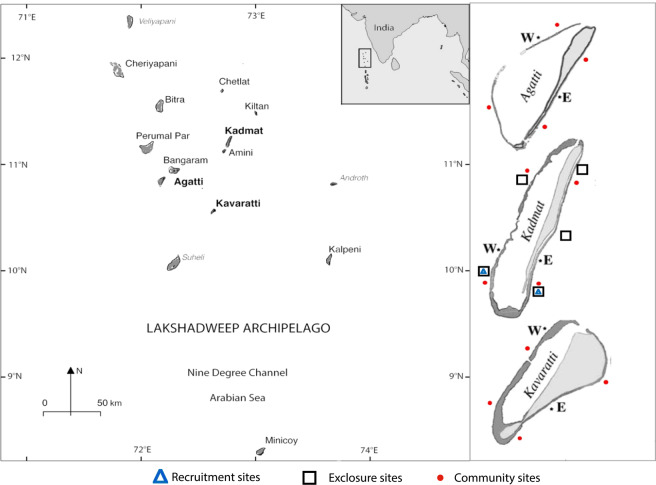
Map of Lakshadweep: Three atolls (*Agatti, Kadmat* and *Kavaratti)* showing sampled reefs in two exposure regimes: sheltered (East, “E”) and exposed (Western, “W”), n = 12). Sites for the exclosure experiments are marked with a square symbol, sites of UVC surveys marked in red dots and those of surgeon fish recruitment survey are marked with blue triangle in *Kadmat* atoll.

The study was conducted between November 2013 and February 2015 in the Lakshadweep Archipelago (8°N–12°N and 71°E–74°E, Fig. 1). The atolls have distinct windward (west, exposed) and leeward (east, sheltered) aspects in relation to the south-west monsoon^30^, which plays a strong role in shaping coral and fish assemblages^24,29,31–33^. Given these clear differences, we expected fish herbivore composition, biomass, density, herbivore activity and function (algal control) to differ between reef exposures.

To understand how herbivore fish species composition, abundance and feeding behaviour varies between exposed and sheltered reefs, we compared 12 reefs (6 exposed and 6 sheltered reefs) distributed across three atolls (Agatti, Kadmat and Kavaratti, Fig. 1). Sites had a minimum distance of 5 km between them, based on the typical home range of the largest herbivores in the community^34,35^. To determine the relative strength of algal growth versus herbivory between exposed and sheltered reefs, we deployed herbivory exclosures in 2 exposed and 3 sheltered reefs for a period of three months (Fig. 1). In addition, to establish the role of early life history processes in determining herbivore populations, we took opportunistic advantage of a multi-species herbivore recruitment event in one exposed and one sheltered reef (see Fig. 1 in ref. ^36^). All sampling was conducted on the outer forereef of the atoll. The outer forereef typically stretches as a flat for a few 100  meters, before transitioning sharply to a steep slope. Our sampling was limited to the outer forereef flat, at a depth of 5–8 meters. The study was conducted during the post-monsoon season (from November to April) when the waves were less intense.

### Herbivore species composition and abundance

We used visual censuses to estimate the abundance of herbivorous fish species. There has been a recent re-evaluation of the nutritional roles of nominal herbivores on coral reefs. Some species, including numerically important species like *Ctenochaetus striatus* may more likely be detritivores^37^, even if they may functionally still play an important role in herbivory^38^. Similarly, collating feeding behaviour, trophic anatomy and biochemical analysis of diets, Clements *et al*.^39^ suggest that most parrotfish may actually be microphages. For convenience though, we refer to the entire assemblage as herbivores, including browsers (species that consume macroalgae) and grazers (species that consume organic benthic material, algae or otherwise). Based on their feeding, we classified species into five functional guilds (browser, detritivore, excavator, cropper, and scraper, Electronic supplementary material, ESM Table 1). All individuals greater than 5 cm in total length were recorded in 3 stationary point counts of 5 m radius established at 0, 25 and 50 m on each of two randomly laid 50m transects per reef . Each point was sampled for three minutes by a single observer (PR), pooling data from each point along the transect (area sampled = 235.6 m^2^). We used stationary point counts since they are more accurate at estimating densities of reef fish^40^. Surveys were conducted within an hour of the high tide, corresponding to peak foraging time. Fish species were identified (as per Ref. ^41^) and placed into four size categories: 5 to <10 cm, 10 to <30 cm, 30 to <50 cm and >50 cm, chosen for ease of identification in the field. We used the midpoints of each size category (ie. 7.5 cm, 15 cm, 40 cm and 50 cm) to convert fish lengths to biomass using standard allometric conversions: W = a × TL^b^ where *W*, is weight in grams, *TL* is total length, and *a* and *b* are species-specific allometric constants obtained from FishBase^42^. We estimated mean biomass for all herbivores (total biomass) and the five guilds (guild biomass) at each reef by averaging the sum of herbivore biomass in both transects and transforming this value to 100 m^2^ area. To account for differences in the number of individuals at each reef , species richness was rarefied using the package Vegan in R^43^. Species richness was calculated as total species richness (all fish herbivores) and species richness by guild for each transect, averaged for each reef (sheltered & exposed n = 12) and island (n = 3).

### Herbivory rates

To quantify herbivory on algal turfs, we used underwater video cameras (GoPro 3) to measure bites taken by each herbivore species in a 3 m^2^ area in front of the camera (marked using buoys). At all reefs, 3 cameras were located randomly >20 meters apart, recording bite rates for 30 minutes. Videos were analysed to quantify which species and functional guilds contributed to turf herbivory across all reefs (less than 1% of the bites were on erect algae). Herbivory rates were estimated as the total number of bites on turf for each species divided by the total recording time (per minute) in a fixed area (3 meters length and 1 meter width). We could not individually identify individuals within each video, which is why we pooled all bites to obtain species-level totals. In order to estimate total herbivory rates (per minute and area) we added bite rates of each species for each video. The resulting herbivory rates were averaged across the three videos to estimate total and species-specific herbivory rates for each reef (bites min^−1^ m^−2^). In order to obtain per capita herbivory rates, we divided average species-specific bite rates from the three videos by the average abundance of each species (obtained from the transects) per m^2^.

### Impact of reef exposure and herbivore control on algal growth

We used herbivore exclusion cages to determine if algal growth rates and herbivory control varied between reef exposures. We established thirteen exclosures (20 ×20 ×20 cm tall) in shallow locations (5–8 m) at two exposed and three sheltered reefs (2–3 cages per reef, see Fig. 1). Cages were constructed of galvanized mesh (mesh size-2.5 cm) and attached directly to the reef substrate, ensuring that most herbivorous fishes were excluded. Cages were regularly cleaned to reduce fouling-related artefacts like light reduction.

We assessed the ability of herbivores to control algal growth (Herbivory impact). Permanently marked areas of 100 cm^2^ inside, and adjacently outside each cage were scraped clean at the start of the experiment. In most cases, the benthos was dominated by turf algae and crustose coralline algae (CCA). Within the exclosure, scraped areas were located at the centre of the quadrat to avoid cage effects. At the end of the experimental period (~90 days) we measured turf canopy height inside and outside the cage with a millimetre scale at five random points within each of the marked areas. To estimate algal growth, we evaluated differences in canopy height inside the cages (mm month^−1^) in both exposures inside the scraped areas. ‘Herbivory impact’ was estimated as the difference in the rate of algal growth (mm month^−1^) inside and outside the exclosures for each replicate quadrat.

### Impact of reef exposure on herbivore recruitment patterns

In February 2015, we encountered a multi-species surgeonfish mass-recruitment event in Kadmat atoll^36^. We documented the event at two locations, one in each exposure regime, very close to our study sites, see Fig. 1 in ref. ^36^). This served as an ideal opportunity to study recruitment patterns in surgeonfish between exposed and sheltered reefs. Reefs were sampled on three occasions at fortnightly intervals: the day after, 12 and 35 days after the recruitment event. At each reef (5–10 m depth) we randomly sampled 10–12 quadrats (each of 4m^2^) at least 20 m apart, visually estimating the abundance and size (cm) of all settlers (<5 cm) with ‘transitional’ colour patterns^44^ in each quadrat. The most abundant species in the recruitment event were *Ctenochaetus striatus* and *Acanthurus nigrofuscus*, which were also the two most abundant and functionally important surgeonfish species identified in this study (see Results). At the start of the recruitment *C. striatus* recruits had a purple colouration with orange lateral striations while *A. nigrofuscus* recruits were evenly brown in colour. By the 35^th^ day post-recruitment, recruits had developed typical adult colours, had grown to >5 cm, and were therefore assumed to be settled juveniles in the reef community.

### Statistical analysis

We used linear mixed-effect models (GLMMs) to model variation in the response variables fish biomass (kg.100 m^−2^), species richness (richness.transect^−1^) and bite rates (bites.min^−1^.m^−2^). This was done for i. all herbivores and ii. each functional guild independently (browser, detritivore, excavator, cropper, and scraper)^26^. We used reef exposure (sheltered, exposed) and atoll as fixed effects and sites nested within atolls as random effects. We began with a global model including both covariates and their interaction terms. We used backward model selection to find the best-fit model. The protocol first finds optimal structures in random effects and then fixed effects by comparing sequentially simplified  models using Likelihood ratio-tests and diagnostic plots of residuals^45^. Bite rates and species richness were square root transformed while biomass was log transformed to normalize the variables. Standard residual diagnostics (Q-Q plots, residual vs fits plot) were used to ensure that model assumptions were met. Data from a total of 12 reefs (6 exposed and 6 sheltered) were used in the analysis. All fish transects (n = 2) or videos (n = 3) were averaged at the reef level.

To test the effect of exposure on algal growth rates (mm month^−1^), we conducted a two-factorial ANOVA, with canopy growth rate (mm month^−1^, n = 13 treatments, averaging all canopy heights within marked areas) as the dependent factor, and exclosure (inside/outside) and reef exposure (exposed/sheltered) as independent factors. The difference in algal growth rates inside and outside the cages in the ANOVA was used as a measure of ‘herbivory impact’.

To assess differences in recruit densities at the exposed and sheltered site in Kadmat over three time periods (Day 1, 12 and 35 after recruitment), we used non-parametric bootstrapping (sampling with replacement, 1000 iterations), to compute 95% Confidence Intervals (CI) of mean recruit densities at each site. Non-overlapping CIs were taken to indicate significant differences in group means^46^. We present mean recruit densities (recruits m-^2^) with bootstrapped (n = 1000 samples, with replacement) 95% CIs on days 1, 12 and 35 since recruitment for comparison between the sheltered and exposed reef.

All analyses were conducted using the statistical software R version 3.0.0^47^. ANOVAs and GLMMs were conducted by using the package MASS^48^ and nlme^49^. Bootstrapping was conducted using package rcompanion^50^ and boot^51^. Data were cleaned organised and plotted using package tidyr and ggplot2^52,53^.

## Results

### Effect of reef exposure on herbivore community composition and herbivory rates

**Figure 2:**
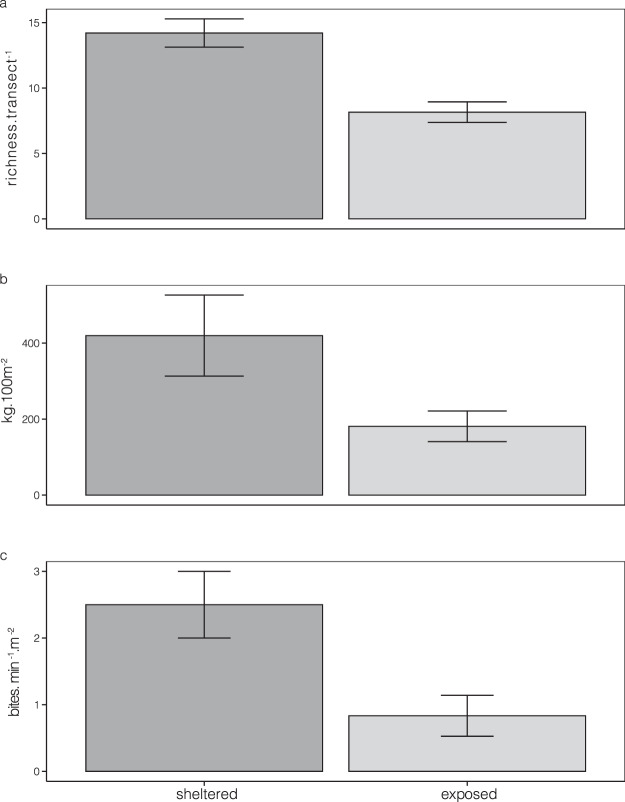
Total fish composition and behaviour between exposure regimes. Differences (Mean ± SE). in (**a**). Total rarefied species richness (richness. transect^−1^), (**b**). Total Biomass (kg.100 m^−2^) and (**c**). Total Bite rates (bites. min^−1^.m^−2^) of all herbivorous fish, between exposure regimes (sheltered and exposed reefs, n = 12). Total transect area is 235.6m^2^ (see methods).

Total herbivore species richness, biomass and bite rates were two times higher on sheltered than exposed reefs (Fig. 2, Table 1). Exposure explained ~60% of total variation in total richness and bite rates. Conversely, exposure explained only 33% of the total variation in biomass, where considerable atoll and site-wise variation was observed (Table 1b).

**Table 1:** Differences in herbivore composition and herbivore function between exposure regimes.

Model	Random effects			Fixed effects					Goodness of fit	
	atoll	site	residual		coefficient	se	t	p	R^2^m	R^2^c
a. Species richness richness.transect^1^ ~exposure random= atoll/site	0.665	1.963	0.675	Intercept	13.666	0.930	14.683	<0.005	0.673	0.968
				Exposure (Exposed)	−6	1.198	−5.004	**0.001**		
b. Biomass log(kg.100 m^−2^) ~exposure random= atoll/site	0.298	0.532	0.203	Intercept	5.889	0.289	20.336	<0.005	0.323	0.932
				Exposure (Exposed)	−0.851	0.329	−2.588	**0.032**		
c. Bite rates sqrt(bites.min^−1^.m^−2^) ~ exposure random= atoll/site	0.383	0.352	0.154	Intercept	1.532	0.271	5.647	<0.005	0.515	0.608
				Exposure (Exposed)	−0.796	0.222	−3.584	**0.007**		

Linear Mixed models results for a. Total rarefied species richness (richness. transect^−1^), b. Total biomass (kg. 100 m^−2^) and **c**. Total bite rates (bites. min^−1^.m^−2^) of the herbivore community. Response variables are modelled with exposure and atoll as fixed effects and sites within islands as random effects. The best models are presented according to a backward model selection procedure based on sequential Likelihood-Ratio tests (see methods). In addition to random and fixed effect coefficients and SE, goodness-of-fit (GOF) measures are provided for the model: marginal R^2^ (R^2^m), measuring variation explained by fixed effects only, and conditional R^2^ (R^2^c), measuring variation explained by both fixed and random effects. Bite rates is square root transformed and biomass is log transformed.

**Figure 3:**
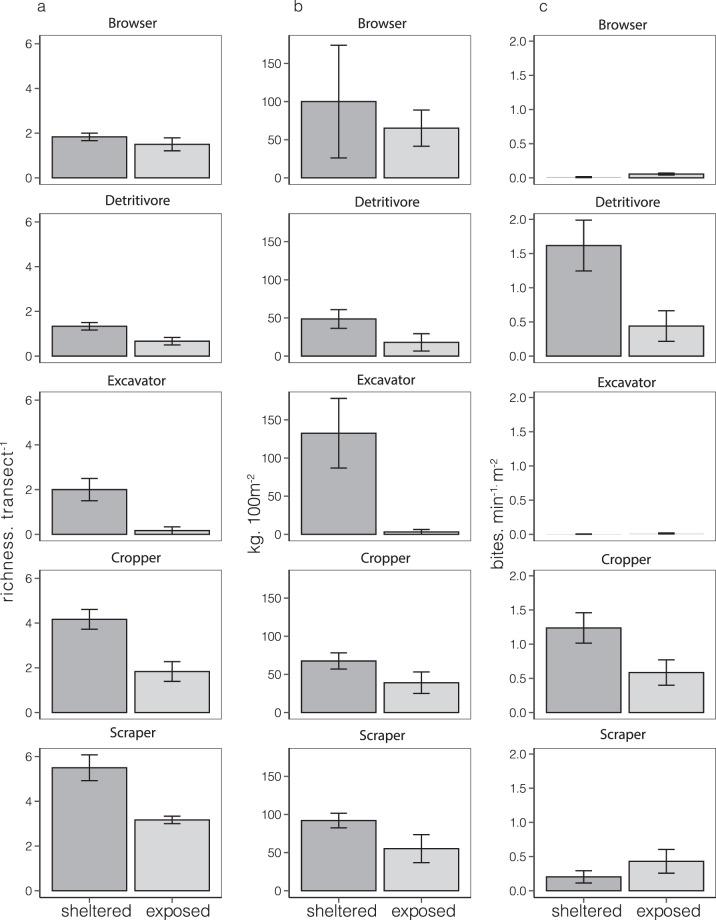
Fish functional guild composition and behaviour between exposure regimes: Differences (Mean ± SE). in (**a**). Rarefied species richness (richness. transect^−1^) (**b**). Biomass (kg.100 m^−2^) and (**c**). Bite rates (bites. min^−1^.m^−2^) between exposure regimes (sheltered and exposed reefs, n = 12). Herbivores are classified into 5 guilds as: Browsers, Croppers, Detritivores, Excavators and Scrapers (ESM Table 1). Total transect area is 235.6m^2^ (see methods).

Species richness of three functional guilds (croppers, excavators and scrapers) varied significantly with reef exposure and was nearly two times higher on sheltered than exposed reefs (Table 2a, Fig. 3a). Species richness of browsers and detritivores was low (~2 species. transect ^−1^) and did not vary significantly with exposure regimes across the three islands, potentially owing to high within-site differences (Table 2a).

**Table 2:** Differences in functional guild composition and herbivore function between exposure regimes.

Model	Random effects			Fixed effects					GOF	
	atoll	site	residual		coefficient	se	t	p	R^2^m	R^2^c
**A Guild richness**										
Browser	0.176	0.429	0.154	Intercept	1.833	0.212	8.629	0.000	0.303	0.914
				Exposure (Exposed)	−0.333	0.264	−1.265	0.242		
Cropper	0.554	0.667	0.275	Intercept	4.167	0.435	9.578	<0.005	0.641	0.967
				Exposure (Exposed)	−2.333	0.417	−5.600	**<0.005**		
Excavator	0.353	0.715	0.267	Intercept	2.000	0.373	5.367	0.001	0.564	0.955
				Exposure (Exposed)	−1.833	0.441	−4.158	**0.003**		
Scraper		1.039	0.389	Intercept	5.500	0.453	12.131	0.000	0.546	0.987
				Exposure (Exposed)	−2.333	0.641	−3.639	**0.005**		
Detritivore		0.483	0.181	Intercept	1.333	0.211	6.325	<0.005	0.312	0.915
				Exposure (Exposed)	−0.667	0.298	−2.236	0.049		
**B. Guild biomass**										
Browser		0.961	0.360	Intercept	3.230	0.726	4.448	0.004	0.665	0.958
				Exposure (Exposed)	−2.131	1.027	−2.075	0.083		
				Atoll (Kadmat)	−1.106	1.027	−1.077	0.323		
				Atoll (Kavaratti)	1.620	1.027	1.577	0.166		
				Exposed*Kadmat	4.351	1.452	2.996	**0.024**		
				Exposed*Kavaratti	1.954	1.452	1.346	0.227		
Cropper		0.258	0.096	Intercept	1.131	0.159	7.117	<0.005	0.777	0.972
				Exposure (Exposed)	−0.876	0.159	−5.513	**0.001**		
				Atoll (Kadmat)	0.275	0.195	1.411	0.196		
				Atoll (Kavaratti)	0.549	0.195	2.822	**0.022**		
Excavator		1.660	0.622	Intercept	4.498	1.024	4.394	0.002	0.546	0.944
				Exposure (Exposed)	−3.476	1.024	−3.395	**0.009**		
				Atoll (Kadmat)	−1.478	1.254	−1.179	0.273		
				Atoll (Kavaratti)	−0.117	1.254	−0.093	0.928		
Scraper		0.635	0.238	Intercept	3.800	0.392	9.690	<0.005	0.524	0.941
				Exposure (Exposed)	−0.903	0.392	−2.303	**0.050**		
				Atoll (Kadmat)	0.850	0.480	1.769	0.115		
				Atoll (Kavaratti)	1.225	0.480	2.550	**0.034**		
Detritivore		1.263	0.477	Intercept	2.804	0.783	3.583	0.007	0.436	0.930
				Exposure (Exposed)	−1.871	0.783	−2.391	**0.044**		
				Atoll (Kadmat)	1.601	0.958	1.671	0.133		
				Atoll (Kavaratti)	0.873	0.958	0.911	0.389		
**C. Bite rates**										
Browser	0.037	0.077	0.028	Intercept	0.066	0.040	1.639	0.140	0.446	0.944
				Exposure (Exposed)	0.156	0.048	3.278	**0.011**		
Cropper		0.249	0.093	(Intercept)	1.089	0.109	10.019	<0.005	0.343	0.919
				Exposure (Exposed)	−0.369	0.154	−2.400	**0.037**		
Excavator		0.070	0.026	(Intercept)	0.015	0.037	0.408	0.693	0.178	0.898
				Atoll (Kadmat)	0.030	0.053	0.576	0.578		
				Atoll (Kavaratti)	0.081	0.053	1.531	0.160		
Scraper	0.100	0.261	0.092	Intercept	0.382	0.127	3.001	0.017	0.116	0.912
				Exposure (Exposed)	0.206	0.160	1.283	0.236		
Detritivore		0.425	0.159	Intercept	1.215	0.186	6.550	<0.005	0.413	0.927
				Exposure (Exposed)	−0.731	0.262	−2.784	**0.019**		

Linear Mixed models results for a. Rarefied species richness (richness. transect^−1^), b. Biomass (kg. 100 m^−2^) and c. Bite rates (bites. min^−1^.m^−2^) of functional guilds (Browsers, Croppers, Detritivores, Excavators and Scrapers – see ESM Table 1). Response variables are modelled with exposure and atoll as fixed effects and sites within islands as random effects. The best models are presented according to a backward model simplification procedure based on sequential Likelihood-Ratio tests (see methods). In addition to random and fixed effect coefficients and SE, goodness-of-fit (GOF) measures are provided for the model: marginal R^2^ (R^2^m), measuring variation explained by fixed effects only, and conditional R^2^ (R^2^c), measuring variation explained by both fixed and random effects. Bite rates and species richness are square root transformed and biomass is log transformed.

The biomass of most guilds (scrapers, croppers, excavators and detritivores) varied significantly with exposure regimes and atolls (Table 2b, Fig. 3b). In some groups like large excavators, biomass was nearly ten times higher in sheltered reefs and as low as 4 kg 100 m^−2^ in exposed reefs. Scrapers and croppers had higher biomass in sheltered reefs of Kavaratti compared to other atolls. Browsers did not vary in biomass by exposure, except in Kadmat (Table 2b), where the difference in biomass was nearly an order of magnitude between exposed and sheltered reefs. Nearly 20% of variation in guild biomass was explained by within site differences (Table 2b).

Despite large differences in biomass, bite rates of only few functional guilds varied with exposure (Table 2c). Interestingly, browsers had higher bites rates in exposed reefs, while croppers and detritivores had higher bite rates in sheltered reefs. Croppers and detritivores had the highest herbivory rates in sheltered reefs [Between 1.5–2 bites min^−1^ m^−2^] compared to browsers, scrapers and excavators [less than 1 bites min^−1^ m^−2^]. Overall, exposure explained a very small portion of total variation in the models (15–45%), which showed large within site and within atoll variation (Table 2c).

### Effect of reef exposure on species-specific abundance, herbivory and per capita bite rates

**Figure 4:**
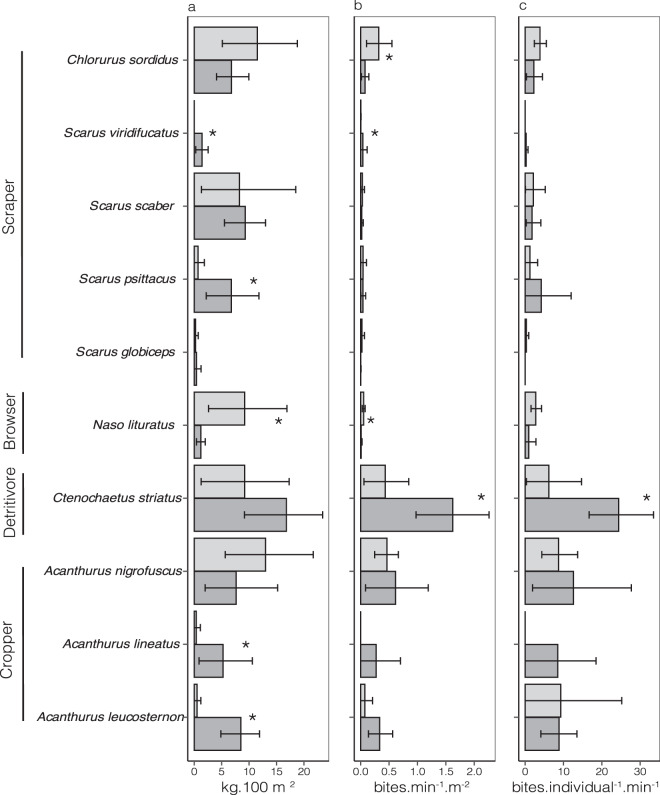
Fish species biomass, herbivory and behaviour between exposure regimes of ten herbivore species that contribute to 95% of total bites across sites (n = 12). (**a**) Difference (Mean ± 95% CI) in total biomass (kg.100 m^−2^) (**b**). Difference (Mean ± 95% CI) in total herbivory (bites. min^−1^. m^−2^) and (**c**). Difference (Mean ± 95% CI) in herbivore behaviour (bites. individual^−1^_._ min^−1^) between exposures (sheltered and exposed reefs, n = 12). Asterisks * indicate significant differences between group means (non-overlapping CIs).

Of the ten most active (contributing to 95% of total bites) herbivore species, five species *Ctenochaetus striatus, Acanthurus nigrofuscus, Acanthurus lineatus, Acanthurus leucosternon* and *Chlorurus sordidus* contributed more than 90% of the total bites (Fig. 4b). Of these *C. striatus* was the most active (Fig. 4a), representing 50% of the total bites (Fig. 4b), followed by *A. nigrofuscus*, which contributed 20% of the total bites (Fig. 4b). Total bite rates of *C. striatus* were clearly higher in sheltered reefs compared to exposed ones. In contrast, *A. nigrofuscus* contributed equally to total bites on both aspects (Fig. 4b). Biomass of most scrapers and cropper species was higher in sheltered reefs, however biomass of the browser *Naso.lituratus* was higher in exposed reefs (Fig. 4a). Interestingly although there was no difference in total biomass between exposure regimes, per capita herbivory rate (bite rates divided by abundance) of the detritivore (*C. striatus*) was consistently higher in sheltered reefs in all three islands (Fig. 4c) suggesting a higher activity in sheltered reefs. For all other species, patterns of per capita herbivory did not differ with exposure regimes (Fig. 4c).

### Effect of reef exposure and herbivore control on algal growth (canopy height, mm month-1)

**Figure 5:**
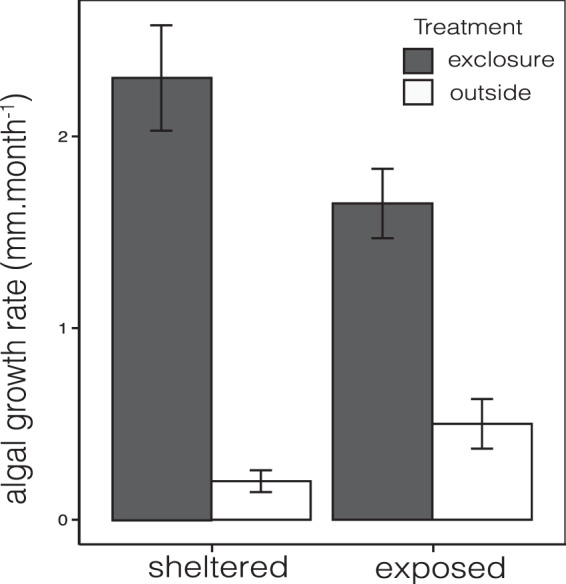
Exposure-mediated algal control by fish herbivores: Differences (Mean ± SE) in algal growth rate (mm month^−1^) inside and outside herbivore exclosures (n = 13) established in sheltered and exposed reefs (n = 5) in *Kadmat atoll*.

Over three months of the experimental exclusion of herbivores, growth rates (mm month^−1^) of turf algae differed significantly inside and outside exclosures and between reef exposures (F_1/19_ = 29.15, p = <0.005, R^2^ = 0.821, Table 3, Fig. 5). Herbivory impact (difference in growth rates of turf algae between exclosures vs outside exclosures) was nearly two times higher in sheltered reefs (Fig. 5, Table 3).

**Table 3:** The ability of exposure to mediate algal control by herbivores: ANOVA results of algal growth rate (mm.month ^−1^) inside and outside exclosures (n = 13), in sheltered and exposed reefs (n = 5) in Kadmat atoll.

Analysis of variance	Parameters	Df	SumS	MeanSS	F	Pr(>F)
Algal growth rate ~ Exclosure x Exposure	Exclosure	1	13.1	13.1	79.338	<0.0001
	Exposure	1	0.126	0.126	0.763	0.394
	Exposure x Exclosure	1	1.214	1.214	7.35	**0.014**
	Residuals	19	3.137	0.165

### Surgeonfish recruitment patterns

**Figure 6:**
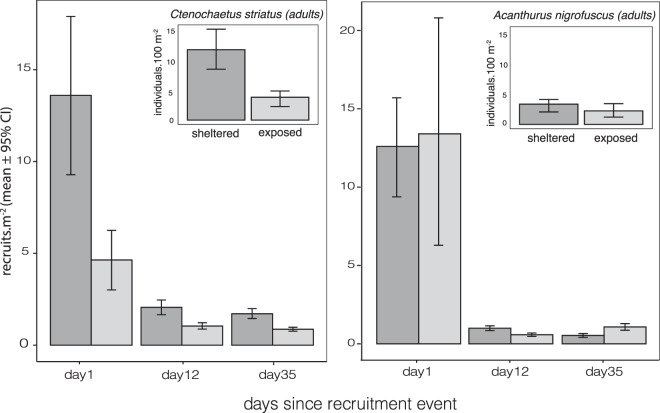
Differences in recruit density (recruits m^−2^) of the two most abundant herbivore species (a. *Ctenochaetus striatus*, b. *Acanthurus nigrofuscus*) between exposure regimes (sheltered and exposed reef, n = 2) in Kadmat atoll. Sampling was conducted on day 1, 12 and 35 after the recruitment event (February 14^th^ 2015). Recruits were classified as individuals <5 cm, showing intermittent colour forms. The inset graphs show adult abundance (individuals. 100 m^−2^) of the two species at the same sites from this herbivore survey. Adults were identified as individuals >5 cm showing fully developed adult colouration.

On the day of the recruitment event, mean densities of recruits (recruits m^−2^) of *Ctenochaetus striatus* and *Acanthurus nigrofuscus* were higher on the sheltered than the exposed reef (Fig. 6a,b). There was a 6–12 fold decline in recruit densities of both *C. striatus* and *A. nigrofuscus* within 12 days since the initial recruitment event (Fig. 6a,b). Within 35 days of recruitment, the species showed contrasting density patterns in sheltered and exposed reefs; *C. striatus* settlers had twice the density on the sheltered reef compared to the exposed reef (Fig. 6a), *A. nigrofuscus* settlers had higher densities on exposed reefs compared to the sheltered reef (Fig. 6b). By day 35, differences in recruit densities matched differences in adult densities of the two species respectively in Kadmat atoll (Fig. 6a,b, insets).

## Discussion

Abiotic factors set the operating space within which ecological interactions occur. By determining which species, each according to its coping ability, can occupy an area, and by limiting their numbers, physical exposure can strongly mediate ecosystem functions^11^. In Lakshadweep, the distinct exposure regimes created by the five-month long southwest monsoon results in a clear difference in the distribution of herbivory on these reefs. Total herbivore activity (bite rates) was substantially reduced in exposed reefs linked mostly to a strong environmental filtering of species composition, abundance and species-specific feeding behaviour – a filtering that for some critical herbivore species potentially begins with differential recruitment and post-recruitment processes between exposures. The bulk of this herbivory (>90% of total bites) was contributed by just a few species, most of them laterally flattened surgeonfish that may have found it more difficult accessing wave-battered shallow reefs^26^.

There were strong compositional differences in the herbivore assemblage between sheltered and exposed aspects. Overall, species richness, abundance, biomass and herbivore activity at exposed reef sites were over two times lower than in sheltered sites. Exclosure experiments confirmed this trend, with exposed reefs being less controlled by herbivores than sheltered reefs; primary production was similar between aspects but not herbivore pressure. The biggest difference in terms of biomass and abundance between exposure regimes was in the number of large excavators, detritivores, croppers and scrapers; of these, detritivores and croppers contributed by far the most to total foraging activity. Even more striking was that, despite the large diversity of herbivores observed on the reefs, only five species were responsible for more than 90% of total bite rates: *Ctenochaetus striatus, Acanthurus nigrofuscus, Acanthurus lineatus, Acanthurus leucosternon* and *Chlorurus sordidus*. Despite the fact that the biomass of browsers and large excavators was much higher on the reef, the bulk of bite rates were contributed by detritivores and croppers. Of these, *C. striatus* and *A. nigrofuscus* were disproportionately important in their bite activity. While there is some consensus about the role *A. nigrofuscus* plays in controlling turf, the role of *C. striatus* and parrotfishes as herbivores is in active debate, with studies suggesting that, as detritivores and microphages, they may play a relatively small role in consuming algae^37,45^. While our own observations cannot resolve this issue, it is telling that herbivore impact (measured with our cage experiments) was highest at sheltered sites, where the abundance and activity of the detritivore *C. striatus* was highest. As has been suggested by others, *C. striatus* may still play an important functional role in controlling turf independent of its nutritional status^38,54^. Of course, other ‘true’ herbivores like *Acanthurus leucosternon* or *Acanthurus lineatus* could also have contributed to turf control at sheltered sites. Although their total feeding activity was less than *C. striatus*, their larger gape sizes and longer feeding bouts may allow them to exert a disproportionate control. How much of the lack of algal control on exposed fronts has to do with the relative effectiveness of these species in removing algae is difficult to tell from our work but is likely to be high. What is evident though is that exposure serves as an important filter of the herbivore assemblage, potentially mediating the way functions are distributed across the reef.

It has long been recognized that herbivores avoid wave-battered reef zones, concentrating their activity in richer, slightly less turbulent waters^55^. Environmental filtering certainly seems to limit the richness and number of herbivore species on exposed fronts in the Lakshadweep (except for the fusiform species, *C. sordidus*, that had higher abundances at exposed sites, and a mid-water species, *Naso literatus*). In addition, exposure served as a behavioural filter for the most abundant species, *C. striatus*. In fact species-specific per capita bite rates were higher in sheltered aspects compared to exposed ones. Recent work shows that body shape and swimming abilities can be major limitations in wave-exposed fronts with laterally compressed species unable to perform well under these flow conditions, while fusiform species do much better^26^. It must be emphasized that our study was conducted after the monsoons, when conditions were relatively calm on exposed reefs compared to the monsoon period. The behavioural filtering we recorded is likely to be much more pronounced during the 5-month south-west monsoon, something this work was unable to capture. However, our study integrates the year-long consequences of monsoon waves on these reefs, showing that even when exposure differences are not as stark, the effects of the monsoon still linger in the compositional and functional distribution of herbivory across these reefs. Higher herbivory at sheltered sites may in addition reflect fish tracking differences in resource availability (bottom-up control), concentrating their feeding where algal production is highest^56^. However, this does not appear to be the case in Lakshadweep, where only a mild, non-significant effect of algal production was observed between exposures (in contrast, see Refs.^57–59^).

Exposure can directly mediate herbivore abundance by filtering fundamental processes such as fish recruitment and survival^11^. It is rarely possible with observational field studies to identify when differences in species distribution begin – which makes our opportunistic recruitment observations of these species particularly valuable. These differences have to be interpreted with caution given their opportunistic nature. Our observations show that both *C. striatus* and *A. nigrofuscus* recruited in higher densities on a sheltered reef compared to an exposed reef. Recruits may prefer the more stable and complex structure of sheltered reefs, which (as discussed later) is itself driven by exposure regimes^36^. Post recruitment survival was low in both species, but after a month of recruiting to the reef, settler densities already reflected differences in adults for these two species. The few field observations of episodic Acanthurid recruitment report high mortality of *C. striatus* in the days and weeks post recruitment, linked to predation or disease^59,60^. In this study, we observed several successful predation events by predatory fish, which could have contributed to rapid post-recruitment declines. For the functionally key *C. striatus*, settlers were twice as high on the sheltered reef, while *A. nigrofuscus* settlers were higher on the exposed reef. Whatever post-recruitment factors cause differences between aspects (differential mortality, movement, competitive exclusion, habitat choice, diseases, etc.), early life-history processes appear to have long-term consequences for the distribution of populations and functions across the reefscape. Whether these patterns are true of other species in the herbivore assemblage will require more dedicated studies of fish recruitment and survival.

While exposure can mediate function directly by modifying assemblages and populations, it also can have strong indirect effects by modifying the underlying structure on which algae grow, and to which fish recruit and inhabit. Four years after the 2010 mass-bleaching event, reef architecture in the Lakshadweep was altered drastically^28^, leaving some reefs with dead standing coral structures and others flattened to rubble^32^. Facing the brunt of the 5-month long southwest monsoon, exposed shallow sites had very low structure compared to sheltered sites^24,31^. For one, with the loss of structurally complexity, turfs may lose a substantial amount of suitable substrate to grow on. Sheltered areas, already characterised by higher growth, also have more areas to colonise, increasing potential resources for fish. In addition, structure also provides a greater spectrum of niches that species can exploit, reducing competition^61–64^. For instance, Fox and Bellwood^65^ showed that rabbitfish exploited crevice-dwelling algae at structurally complex sites, aided by morphological specialisation (longer, narrower snouts and heads), distinct from other herbivores. Niche separation of this sort allows several sympatric reef herbivores to co-exist in complex habitats, becoming less useful in architecturally simplified reefscapes and resulting in species dropping out of these environments. Apart from influencing resources, structure can also directly influence survival of species. For many benthic fish species, this large-scale erosion of structural complexity can be devastating^66,67^. At low-structured sites, predation rates on recruits and juveniles may be much higher; complex structures can strongly influence fish diversity and herbivory by enhancing refuge^68–70^. Structure could be an important covariate driving the large variance seen between sites in biomass and bite rates. While we have not directly measured if the observed trends are a result of direct or indirect mechanisms of environmental filtering, they are critical to understanding how function is distributed across the reef and warrant further study.

Unpacking the mechanisms of ecosystem function is increasingly relevant and urgent as the tropics become dominated by reefs in a state of constant recovery, affected as they are by increasingly frequent coral mass mortalities^28,71^. A few key herbivores may be the unlikely drivers of this recovery, grazing dead reefs clean of algal turfs, thus facilitating new coral recruitment. To what extent herbivores play that critical role in this system is still to be demonstrated. In other regions where herbivores are scarce, thick epilithic turfs proliferate in the reef and potentially inhibit settlement and outcompete young coral in reefs recovering from mass mortalities^72,73^. However, the oceanic atoll reefs in the Lakshadweep are relatively oligotrophic, resulting in very low algal growth rates (1 mm per month, this study). Despite low productivity of epilithic turfs, post-disturbed reefs in Lakshadweep exhibited a protracted recovery, particularly after the 2010 and 2016 events^28,74,75^, owing to low recruitment success of fast-growing coral species. Herbivory can therefore be crucial to reef recovery even in such nutrient-limited islands. In this study, we show that abiotic conditions (like wave exposure) can place significant natural limits on the ability of key grazers to function as effective agents of algal control, potentially hampering reef recovery at physically extreme environments. Exposure appears to be a universal filter, acting directly and potentially indirectly by modifying the underlying benthic structure of dead reefs, influencing suitable recruitment success of key herbivore species, reducing the richness and number of the herbivore assemblage, together resulting in lower rates of herbivory. Whether exposure additionally mediates coral recruitment and survival remains to be seen. Post-disturbed reefs are our new normal. Finding out how they function, and the limits to their functioning, is critical to exploring ways to reverse their decline.

## Supplementary information

Supplementary Information.

## Data Availability

The datasets generated during and/or analysed during the current study are available from the corresponding author upon request.
