# Supplementary material for: Wave exposure reduces herbivory in post-disturbed reefs by filtering species composition, abundance and behaviour of key fish herbivores
Source: Sci Rep. 2020 Jun 19;10:9854. doi: 10.1038/s41598-020-66475-y (PMC7305165; doi:10.1038/s41598-020-66475-y)
Supplement: Supplementary file 1 — Supplementary Information. [file 41598_2020_66475_MOESM1_ESM.docx]

Electronic Supplementary Material

——————————————————————————————————————————

**Title:** Wave exposure reduces herbivory in post-disturbed reefs by filtering species composition, abundance and behavior of key fish herbivores

**Authors**

Rucha Karkarey†, Pooja Rathod†, Rohan Arthur, Shreya Yadav, Anne Theo, Teresa Alcoverro

†Co leading authors

——————————————————————————————————————————

ESM 1. Functional classification of herbivores species in this study.

Fish species (belonging to family: Scarinae, Siganidae and Acanthuridae) were classified into functional guilds as per the widely accepted classification scheme of Green and Bellwood (2009). This classification is based on a combination of factors like herbivore diet, feeding apparatus morphology, feeding behaviour and feeding impact. Functional groups are embedded within two broad categories of ‘erect macroalgal feeders’ and ‘epilithic algal matrix (EAM) feeders’.

| Functional Groups | Description | Potential impact | Species |
| --- | --- | --- | --- |
| ***1. Erect macroalgae feeders*** | | | |
| **Browsers** | Fish that feed on erect macroalgae >10mm in height^1^. | Limit growth of  Fleshy macroalgae^1^. | *Naso brachycentron*  *Naso tuberosus*  *Naso hexacanthus Naso lituratus Naso thynnoides Naso unicornis* |
| ***2. Epilithic algal matrix (EAM) feeders*** | | | |
| **Detritivores** | Fish that feed on organic matter (including diatoms in sediment and reef surfaces) by typically brushing EAM with highly specialized mouthparts. May consume small filamentous turf algae, along with large amounts of unidentified organic matter and may dislodge algae in the process of feeding^2-4^. | Reduce sediments in EAMs to provide favourable settlement surfaces for benthic organisms. May increase the palatability of EAM to herbivorous reef fishes thereby indirectly supporting reef resilience^2-4^. | *Ctenochaetus strigosus*  *Ctenochaetus striatus* |
| **Large excavators** | Fish that take large, deep bites, on calcareous substratum while feeding. Scarinae greater than 35cm in length were included as large excavators^5^. | Key agents of bioerosion and sediment redistribution on reefs. Regulate benthic biota on reefs. Remove EAM and create bare substrata for potential coral recruitment or aid in coral asexual reproduction by dispersing the dislodged coral fragments^1,5^. | *Chlorurus strongycephalus Chlorurus enneacanthus Cetoscarus bicolor*  *Scarus rubroviolaceus*  *Scarus prasiognathus* |
| **Scrapers** | Fish that forage on EAM and while doing so remove parts of the underlying calcareous substrata (‘scraping’). Scrapers typically leave shallow (<1mm) bite scars on substrate. ‘Large excavators’ less than 35 cm in length were included as small excavators^1,5^. | Creating bare substrate by removing the EAM^1,5^. | *Chlorurus atrilunula Chlorurus capistratoides Chlorurus sordidus* |
| small excavators |  |  |  |
| scrapers |  |  | *Hipposcarus harid, Scarus caudofasciatus  Scarus ghobban Scarus globiceps Scarus niger Scarus psittacus Scarus frenatus Scarus russelli Scarus scaber/oviceps Scarus tricolor Scarus viridifucatus* |
| **Croppers** | Fish that feed on algal turf and sediment to extract detritus, microbes and diatoms. | May limit growth of macroalgae in addition to role of detritivores. | *Acanthurus blochi*  *Acanthurus auranticavus Acanthurus lineatus Acanthurus nigricauda Acanthurus tenneti* |
| croppers/detritivores |  |  |  |
| croppers | Fish that ‘crop’ the upper portions of algae and leave the basal portions intact ^1,5^. | Limit growth of macroalgae^1,5^. | *Acanthurus leucosternon*  *Acanthurus nigrofuscus*  *Acanthurus tominiensis Acanthurus triostegus Acanthurus nigroris,*  *Zebrasoma desjardini, Zebrasoma scopas,*  *Siganus argenteus*  *Siganus stellatus*  *Siganus corallinus* |

References:

^1^ Green, A. L. & Bellwood, D. R. 2009 Monitoring functional groups of herbivorous reef fishes as indicators of coral reef resilience - a practical guide for coral reef managers in the Asia Pacific region. IUCN, Gland, Switzerland. 70 p. (ISBN: 978-28317-1169-0).

^2^Goatley, C. H. R. & Bellwood, D. R. 2010 Biologically mediated sediment fluxes on coral reefs: Sediment removal and off-reef transportation by the surgeonfish Ctenochaetus striatus. *Mar. Ecol. Prog. Ser.* **415**, 237–245. (doi:10.3354/meps08761)

^3^Marshell, A. & Mumby, P. J. 2012 Revisiting the functional roles of the surgeonfish *Acanthurus nigrofuscus* and *Ctenochaetus striatus*. *Coral Reefs* **31**, 1093–1101. (doi:10.1007/s00338-012-0931-y)

^4^Choat, J. H., Robbins, W. D. & Clements, K. D. 2004 The trophic status of herbivorous fishes on coral reefs: II. Food processing modes andtrophodynamics. *Mar. Biol.* **145**, 445–454. (doi:10.1007/s00227-004-1341-7) .

^5^Bellwood, D. R. & Choat, J. H. 1990 A functional analysis of grazing in parrotfishes (family Scaridae): the ecological implications. *Environ. Biol. Fishes* **28**, 189–214. (doi:10.1007/BF00751035).

ESM 2. Bootstrapping results of a. biomass, b. total herbivory and c. herbivore behavior (per capita bite rates) of 10 functionally important herbivorous fish (contributing to 95% of total bites) from figure 4.

|  | **exposure** | **species** | **mean** | **CI low** | **CI high** |
| --- | --- | --- | --- | --- | --- |
| 1. Biomass   (kg.100m^-2^) | exposed | *Acanthurus leucosternon* | 0.521 | 0 | 1.22 |
|  |  | *Acanthurus lineatus* | 0.375 | 0 | 1.12 |
|  |  | *Acanthurus nigrofuscus* | 13.1 | 5.08 | 21.7 |
|  |  | *Chlorurus sordidus* | 11.5 | 4.63 | 18.3 |
|  |  | *Ctenochaetus striatus* | 9.19 | 1.26 | 17.3 |
|  |  | *Naso lituratus* | 9.2 | 2.52 | 17.3 |
|  |  | *Scarus globiceps* | 0.246 | 0 | 0.737 |
|  |  | *Scarus psittacus* | 0.702 | 0 | 1.84 |
|  |  | *Scarus scaber* | 8.24 | 0.898 | 18.5 |
|  |  | *Scarus viridifucatus* | 0 | 0 | 0 |
|  | sheltered | *Acanthurus leucosternon* | 8.51 | 5.07 | 11.9 |
|  |  | *Acanthurus lineatus* | 5.26 | 0.89 | 10.1 |
|  |  | *Acanthurus nigrofuscus* | 7.65 | 1.85 | 14.8 |
|  |  | *Chlorurus sordidus* | 6.78 | 4.1 | 10.3 |
|  |  | *Ctenochaetus striatus* | 16.8 | 10.1 | 23.4 |
|  |  | *Naso lituratus* | 1.22 | 0.417 | 1.96 |
|  |  | *Scarus globiceps* | 0.418 | 0 | 1.25 |
|  |  | *Scarus psittacus* | 6.77 | 2.19 | 12.1 |
|  |  | *Scarus scaber* | 9.32 | 5.78 | 12.9 |
|  |  | *Scarus viridifucatus* | 1.42 | 0.284 | 2.55 |
| B. Total bites (bites.min^-1^.m^-2^) | exposed | *Acanthurus leucosternon* | 0.073 | 0 | 0.209 |
|  |  | *Acanthurus lineatus* | 0 | 0 | 0 |
|  |  | *Acanthurus nigrofuscus* | 0.463 | 0.246 | 0.669 |
|  |  | *Chlorurus sordidus* | 0.318 | 0.105 | 0.552 |
|  |  | *Ctenochaetus striatus* | 0.433 | 0.058 | 0.828 |
|  |  | *Naso lituratus* | 0.053 | 0.028 | 0.080 |
|  |  | *Scarus globiceps* | 0.022 | 0 | 0.065 |
|  |  | *Scarus psittacus* | 0.041 | 0.002 | 0.104 |
|  |  | *Scarus scaber* | 0.029 | 0.003 | 0.066 |
|  |  | *scarus viridifucatus* | 0.003 | 0 | 0.007 |
|  | sheltered | *Acanthurus leucosternon* | 0.334 | 0.12 | 0.574 |
|  |  | *Acanthurus lineatus* | 0.272 | 0 | 0.7 |
|  |  | *Acanthurus nigrofuscus* | 0.614 | 0.107 | 1.17 |
|  |  | *Chlorurus sordidus* | 0.077 | 0.015 | 0.14 |
|  |  | *Ctenochaetus striatus* | 1.62 | 0.963 | 2.23 |
|  |  | *Naso lituratus* | 0.008 | 0 | 0.023 |
|  |  | *Scarus globiceps* | 0.002 | 0 | 0.007 |
|  |  | *Scarus psittacus* | 0.040 | 0.002 | 0.088 |
|  |  | *Scarus scaber* | 0.019 | 0.002 | 0.045 |
|  |  | *scarus viridifucatus* | 0.038 | 0 | 0.114 |
| C. Percapita bites (bites.individual^-1^.min^-1^) | exposed | *Acanthurus leucosternon* | 9.28 | 0 | 25.2 |
|  |  | *Acanthurus lineatus* | 0 | 0 | 0 |
|  |  | *Acanthurus nigrofuscus* | 8.7 | 4.04 | 13.6 |
|  |  | *Chlorurus sordidus* | 3.87 | 2.4 | 5.34 |
|  |  | *Ctenochaetus striatus* | 6.18 | 0.345 | 14.3 |
|  |  | *Naso lituratus* | 2.74 | 1.49 | 4.3 |
|  |  | *Scarus globiceps* | 0.311 | 0 | 0.932 |
|  |  | *Scarus psittacus* | 1.25 | 0 | 3.27 |
|  |  | *Scarus scaber* | 2.14 | 0.067 | 5.31 |
|  |  | *Scarus viridifucatus* | 0 | 0 | 0 |
|  | sheltered | *Acanthurus leucosternon* | 8.82 | 4.16 | 13.8 |
|  |  | *Acanthurus lineatus* | 8.51 | 0 | 18.5 |
|  |  | *Acanthurus nigrofuscus* | 12.6 | 1.92 | 28.1 |
|  |  | *Chlorurus sordidus* | 2.27 | 0.304 | 4.5 |
|  |  | *Ctenochaetus striatus* | 24.4 | 16.1 | 33.3 |
|  |  | *Naso lituratus* | 0.938 | 0 | 2.81 |
|  |  | *Scarus globiceps* | 0 | 0 | 0 |
|  |  | *Scarus psittacus* | 4.2 | 0 | 12 |
|  |  | *Scarus scaber* | 1.79 | 0.297 | 4.07 |
|  |  | *Scarus viridifucatus* | 0.247 | 0 | 0.741 |
